# Editorial: Real-world surgical treatment of thoracic cancer in the era of precision medicine

**DOI:** 10.3389/fsurg.2022.928131

**Published:** 2022-06-28

**Authors:** Guobang Wei, Yongbing Chen

**Affiliations:** Department of Thoracic Surgery, The Second Affiliated Hospital of Soochow University, Suzhou, China

**Keywords:** thoracic cancer, surgery, real-world evidence, evidence-based medicine, prognosis

**Editorial on the Research Topic**

Real-world surgical treatment of thoracic cancer in the era of precision medicine

Surgical resection has been the first choice for treatment of early-stage thoracic cancer, mainly including lung and esophageal malignancies. Nowadays, the advent of the era of precision medicine, has highlighted the significant roles of evidence-based practice, not only applicable for surgical procedures, but also for perioperative management (1). In this section of “Frontiers in Surgery”, several studies were included which might facilitate the perfection of real-world surgical treatment.

Conventionally, lobectomy and systematic lymphadenectomy is the standard of care for the management of early-stage non-small cell lung cancer (NSCLC) (2, 3). However, numerous factors might exert impacts on the prognosis of patients with stage IA NSCLC receiving lobectomy, such as tumor pathological type and extent of lymph node dissection. Previously, Long et al. (2021) proposed that the order of vascular processing in lobectomy might become a prognostic factor that should not be ignored. More specifically, although there was no significant difference in recurrence rate between the vein-first group and the artery-first group, the vein-first group had better overall survival (OS) and disease-free survival (DFS), especially in the squamous cell carcinoma (LUSC) subgroup and the stage I-II subgroup. Similarly, in the study by Wei et al. (4), the vein-first group exhibited significantly better outcomes than the artery-first group for 5-year OS (73.6% vs 57.6%; *P* = 0.002), DFS (63.6% vs 48.4%; *P* = 0.001), and lung cancer-specific survival (LCSS) (76.4% vs 59.9%; *P* = 0.002). In the context of pulmonary function reserved as an important indicator, sublobar resection, including segmentectomy and wedge resection, has captured increasing attention in recent years which has shown non-inferiority to lobectomy for patients with stage T1a-bN0M0 NSCLC (5, 6). In our section, Hao et al. (2021) suggested that tumor size should be taken as a critical factor for surgical decision-making. In their study, segmentectomy was associated with better OS in patients with NSCLC ≥10 mm and ≤20 mm than wedge resection. Nonetheless, segmentectomy did not exhibit advantages in survival compared with wedge resection in patients with NSCLC ≤10 mm. In addition, it was observed that small-sized (≤20 mm) LUSC was associated with worse OS but not LCSS compared with lung adenocarcinoma. In other word, their findings indicated that surgical procedures and intraoperative manipulation should be personalized based on histology and tumor size.

For patients on whom intentional lobectomy are performed, video-assisted thoracic surgery (VATS) and robotic-assisted surgery (RAS) are two prevalent surgical approaches which are increasingly being paid attention to by virtue of the advantages of perioperative recovery (7). Gallina et al. (2021) pointed out that compared with open surgery, VATS and RAS could effectively reduce the incidence of postoperative complications, while the lymph nodes could be effectively dissected as well. They also found that the percentage of mediastinal lymph node metastasis and the number of lymph nodes dissected in the RAS group were significantly higher than those in the VATS group. More interestingly, the ratio of the number of dissected lymph nodes to the number of metastatic lymph nodes was significantly lower in the VATS group and thoracotomy group compared with the RAS group. The limitation of surgical field and operation space of VATS might account for such a phenomenon. Notably, the aforementioned limitations of VATS did not convert to survival disadvantages, while RAS might bring additional financial burdens to patients (8). In a word, although RAS have been more and more popular and exhibiting advantages in intraoperative manipulation and postoperative recovery, VATS has remained irreplaceable in chest surgery nowadays. More studies should be launched to investigate the pros and cons of RAS.

With accumulating evidences, the advantages of VATS are not only reflected in improving survival expectations of tumor patients (9), but also in having a favorable impact on the postoperative recovery of patients. In our section, Aeschbacher et al. (2021) reported that blood loss >100 ml (*P *= 0.029, HR 2.70) and open surgery (*P *= 0.032, HR 2.37) are independent risk factors for surgical site infections (SSI). SSI occurred much more frequently in open surgery than in VATS approach, and SSI was positively associated with significantly longer hospital stay (10). Undeniably, thoracotomy is currently preferred in the case of intraoperative complications or emergent events with extremely low probabilities, including major vascular injury, calcified lymph nodes around the hilum and dense adhesions. In other word, the studies in our section consistently highlighted the predominant role of VATS in lung cancer surgery.

Hitherto, the treatment of advanced-stage NSCLC patients with distant metastasis has been complex and highly personalized. Previous studies have indicated that systemic chemotherapy or targeted therapy instead of surgery should be recommended for NSCLC patients with malignant pleural dissemination (PD) (11). Sawabata et al. (12) even suggested that tumor resection brought no obvious benefit to postoperative survival of patients who have developed PD. However, Fan et al. (2021) observed that patients who underwent surgical resection of primary tumors had longer progression-free survival (PFS) (19.0 vs. 10.0 months, *P *< 0.0001) and OS (48.0 vs. 33.0 months, *P *< 0.0001) than patients who underwent pleural biopsy alone, suggesting that NSCLC patients with pleural metastasis could still benefit from surgical resection of primary tumors. In addition, postoperative targeted therapy and tumor <3 cm were also favorable prognostic factors, and the survival rate of patients receiving targeted therapy was significantly higher than those without (13). In a large cohort analysis of lung cancer patients with brain metastases, He et al. (2021) proposed that patients who received brain therapy before surgery for primary lung tumors might have a better prognosis, irrespective of the treatment modality on the metastasis site. Furthermore, patients who received brain surgery plus radiotherapy followed by primary lung tumor resection had the best survival expectation. The aforementioned studies indeed shed light on the potential therapeutic scheme of NSCLC patients with M1 disease.

In addition, our section also included some reports on surgical techniques Chen et al. (2021). For instance, reconstruction of the right gastroepiploic vessel may solve the awkward situation of injury of the right gastroepiploic artery and vein during the esophagectomy. Chen et al. (2021) highlighted two key technical points as key resolutions: (1) Immediate reconstruction of the right gastroepiploic artery (RGEA) and right gastroepiploic vein (RGEV) and long-term maintenance of the blood flow effectively; (2) Effective tension reduction of gastric conduit anastomosis and vascular anastomosis.

In a word, this section is intended to be the beginning of a small step towards precision medicine in the field of thoracic cancer, which needs further real-world evidences as stepping stones.
